# A New Eyeless Species of *Micranops* Cameron 1913 from Bolivia (Coleoptera: Staphylinidae: Paederinae)

**DOI:** 10.1007/s13744-023-01106-5

**Published:** 2023-12-14

**Authors:** Yoan Camilo Guzman, Dagmara Żyła

**Affiliations:** 1 Museum and Institute of Zoology, Polish Academy of Sciences, Warsaw, Poland; 2 Museum of Nature Hamburg, Leibniz Institute for the Analysis of Biodiversity Change, Hamburg, Germany

**Keywords:** Endogean way of life, Anophthalmy, Taxonomy, Museum collection

## Abstract

This work adds a new eyeless species of the genus *Micranops* Cameron, 1913 (Staphylinidae, Paederinae, Scopaeina) to the endogean Coleoptera of the Bolivian fauna. The loss of eyes is usually associated with adaptations to subterranean habitats, although *M. bolivianus* Guzman & Żyła **sp. nov.** was collected from leaf litter. We also report new occurrences for the genus west of the Andes mountain range. Our work increases the number of known Neotropical *Micranops* species to seven and extends the known distribution range for the genus in South America.

**Supplementary Information:**

The online version contains supplementary material available at 10.1007/s13744-023-01106-5.

## Introduction

The genus *Micronops* was erected by Cameron in 1913 for a microphthalmos, flightless species, *M. brunneus,* found in Jamaica (Cameron 1913; Frisch and Oromí 2006). Subsequently, additional species with eyes were incorporated into the genus, as discussed by Frisch and Oromi (2006). *Micranops* belongs to the Scopaeina subtribe, and it seems to be closely related to *Scopaeus*, the most species-rich genus of the subtribe (Frisch et al. 2002). The main distinguishing feature that sets *Micranops* apart from *Scopaeus* Erichson, 1839 is the presence of a postorbital furrow from which a trichobothrium emerges, whereas it is absent in *Scopaeus* where the trichobothria are supraorbital (Frisch and Oromí 2006; Herman 2023). *Micranops* is distributed worldwide in the tropics and subtropics, with 33 species described so far (Frisch and Herman 2014; Newton 2021). In the Neotropics, there are six described species: *M. brunneus* Cameron 1913, *M. cameroni* (Blackwelder 1943), *M. volans* (Blackwelder 1943) from Jamaica; *M. chloroticus* (Sharp 1876) from Brazil, *M. myrmecophilus* (Bernhauer 1921) from Argentina, and *M. surinamensis* (Herman 1965) from Surinam. They inhabit a range of altitudes, from high-altitude mountainous habitats to lowlands. They are thermohygrophilous dwellers of humid, sandy soil and usually inhabit the banks of rivers and creeks with sparse pioneer vegetation (Frisch et al. 2002; Frisch and Oromí 2006).

Here, we describe a new species, *Micranops bolivianus* Guzman & Żyła **sp. nov.**, which shows morphological modifications to the endogean way of life such as depigmentation, eyelessness and flightlessness. We also report the presence of the genus from Ecuador and Peru for the first time and add new records of potential females of *M. surinamensis* (Herman 1965) and *M. chloroticus* (Sharp 1876).

## Materials and methods

### Specimen photographs and measurements

We studied eight specimens identified as the new species, all preserved dry and card mounted and coming from the Moravian Museum Brno. Additional material (25 specimens) comes from the Hungarian Natural History Museum, Muséum National d’Histoire Naturelle, and the private collection of Jiří Janák. Label data for specimens are cited verbatim, with a semicolon (;) separating lines of text and a slash (\\) separating labels. Dissections were carried out under an Olympus SZX-7 stereomicroscope. Beetles were boiled in water to soften the tissues. Then, the larger parts were separated and boiled in a 5% KOH solution for maceration of the tissues and cleaning. Then, they were rinsed in water, and the aedeagus was dissected.

Individuals were photographed using a 4 K Ultra-high accuracy microscope VHX-7000 Series by KEYENCE. Scanning electronic micrographs were taken using a Hitachi S-3400N SEM at the Museum and Institute of Zoology Polish Academy of Sciences and a Hitachi TM4000Plus Tabletop Microscope at the Museum of Nature Hamburg, Leibniz Institute for the Analysis of Biodiversity Change (ZMH, LIB). All photos were edited in GIMP 2.10.34 (The GIMP Development Team GIMP 2019), while schematic drawings were made in Inkscape 1.2.2 (Inkscape Project. Inkscape 2020) based on photos and specimens observations.

All measurements were made on the holotype specimen based on photos, using ImageJ V1.53 K (Schneider et al. 2012) and are given in millimetres (mm). The following measurements were made:

Fore-body length – measured from the anterior margin of the clypeus to the posterior end of the elytra.

Head – length measured from the anterior margin of the clypeus to the posterior margin at the midline of the head; width measured at the widest point, including the eyes.

Antenna – length was measured based on separate measurements of all antennomeres, including their stems.

Neck width – measured at the widest point.

Pronotum – length measured at the midline; width measured at the widest point.

Elytra – length measured in two ways: external length (ext) was measured close to the lateral margin of the elytra, while internal length (int) along the suture from the posterior end of the scutellum to the posterior end of the elytra (int); width measured at the widest point of closed elytra; when only one elytron was present, its width was multiplied by two.

Legs – coxal width measured at the base of the articulation; femoral and tibial width measured at the widest part, usually at the most distal end.

### Measurements abbreviations

FBL: Fore-body length
H: Head (length, width)
A: Antennae length
a1-a11: Antennomeres 1–11 (length, width)
NKW: Neck (width)
GL: Gular sutures, separation distance
P: Pronotum (length, width)
E: Elytra (length, width)
PC: Procoxa (length, width)
PF: Profemur (length, width)
PT: Protibia (length, width)
MSC: Mesocoxa (length, width)
MSF: Mesofemur (length, width)
MST: Mesotibia (length, width)
MTC: Metacoxa (length, width)
MTF: Metafemur (length, width)
MTT: Metatibia (length, width)

### Terminology

We mostly used terminology from Bogri et al. (2020) for body parts, while some of the sexual characters of the described species follow Frisch et al. (2002).

### Map

The map was created in RStudio (RStudio Team 2020) using the packages ggplot2 (Wickham 2016) and ggmap (Kahle and Wickham 2013). Coordinates were obtained from the labels when available or using the centroid coordinates of the highest geographical category, such as country, province, or city.

### Depository of the specimens

HNHM Hungarian Natural History Museum, Budapest, Hungary (György Makranczy).

JJRC Private collection of Jiří Janák, Rtyně nad Bílinou, Czech Republic.

MMBC Moravian Museum Brno, Czech Republic (Petr Baňař).

MNHN Muséum national d’Histoire naturelle, Paris, France (Antoine Mantilleri).

UASC Museo de Historia Natural „Noel Kempff Mercado“, Santa Cruz de la Sierra, Bolivia (Julieta Ledezma Arias).

ZMH Zoological Museum Hamburg, Hamburg, Germany (Dagmara Żyła).

## Results

### New species

*Micranops bolivianus* Guzman & Żyła **sp. nov.**

**Fig. 1:**
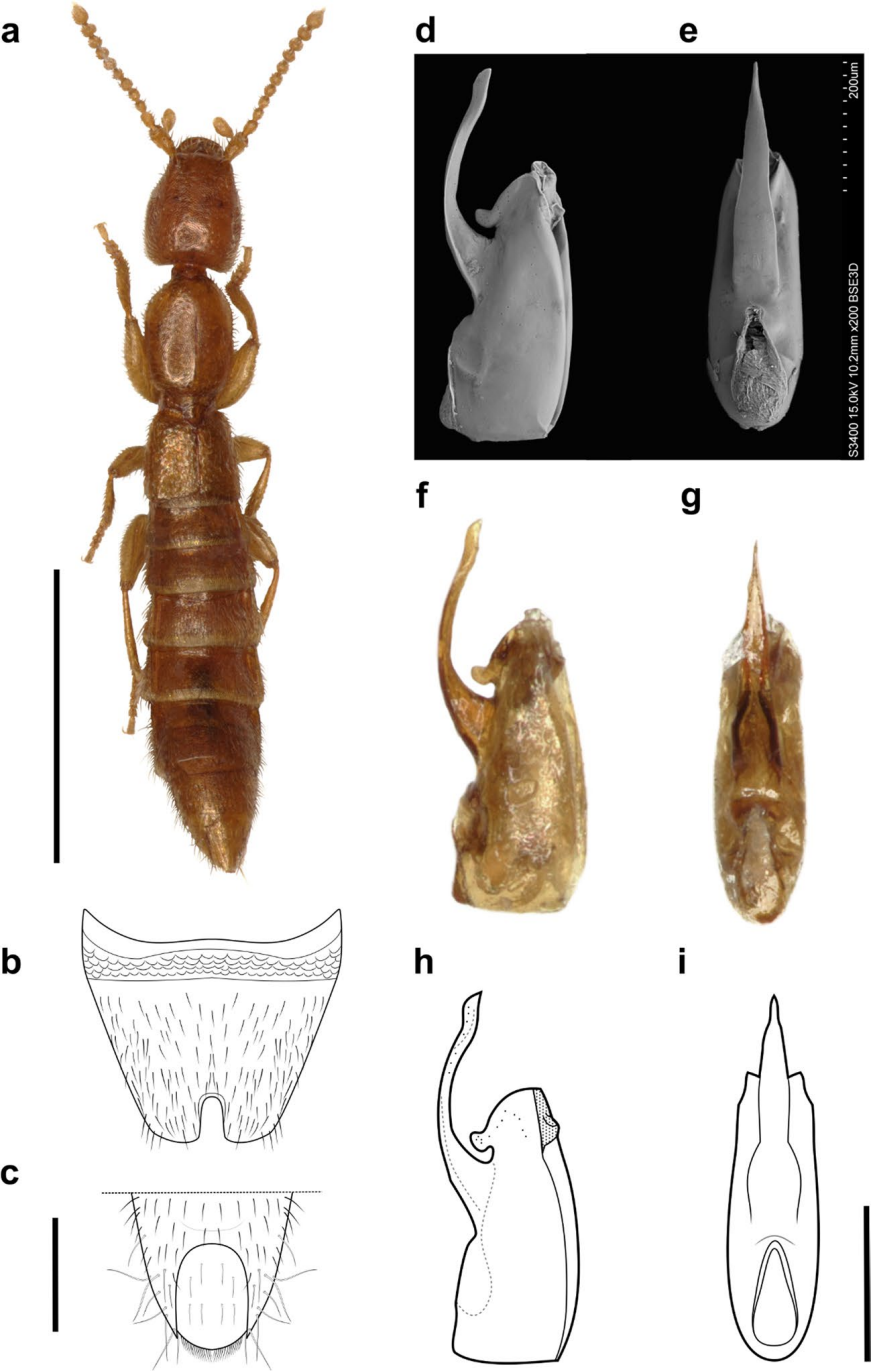
*Micranops bolivianus* Guzman & Żyła **sp. nov. a** Habitus, dorsal. Paratype ♂. Scale bar = 1 mm. **b–c** Scheme of abdominal segments. Scale bar = 0.2 mm. **b** Sternite VIII. **c** Tergite IX–X, posterior margin. **d–i** Aedeagus. Holotype, ♂. **d–e** Scanning electronic micrographs. **d** Lateral view. **f–g** Photographs. **f** Lateral view. **g** Parameral view. **h–i** Schemes. **h** Lateral view. **i** Parameral view. **f–i** Scale bar = 0.25 mm

**Fig. 2:**
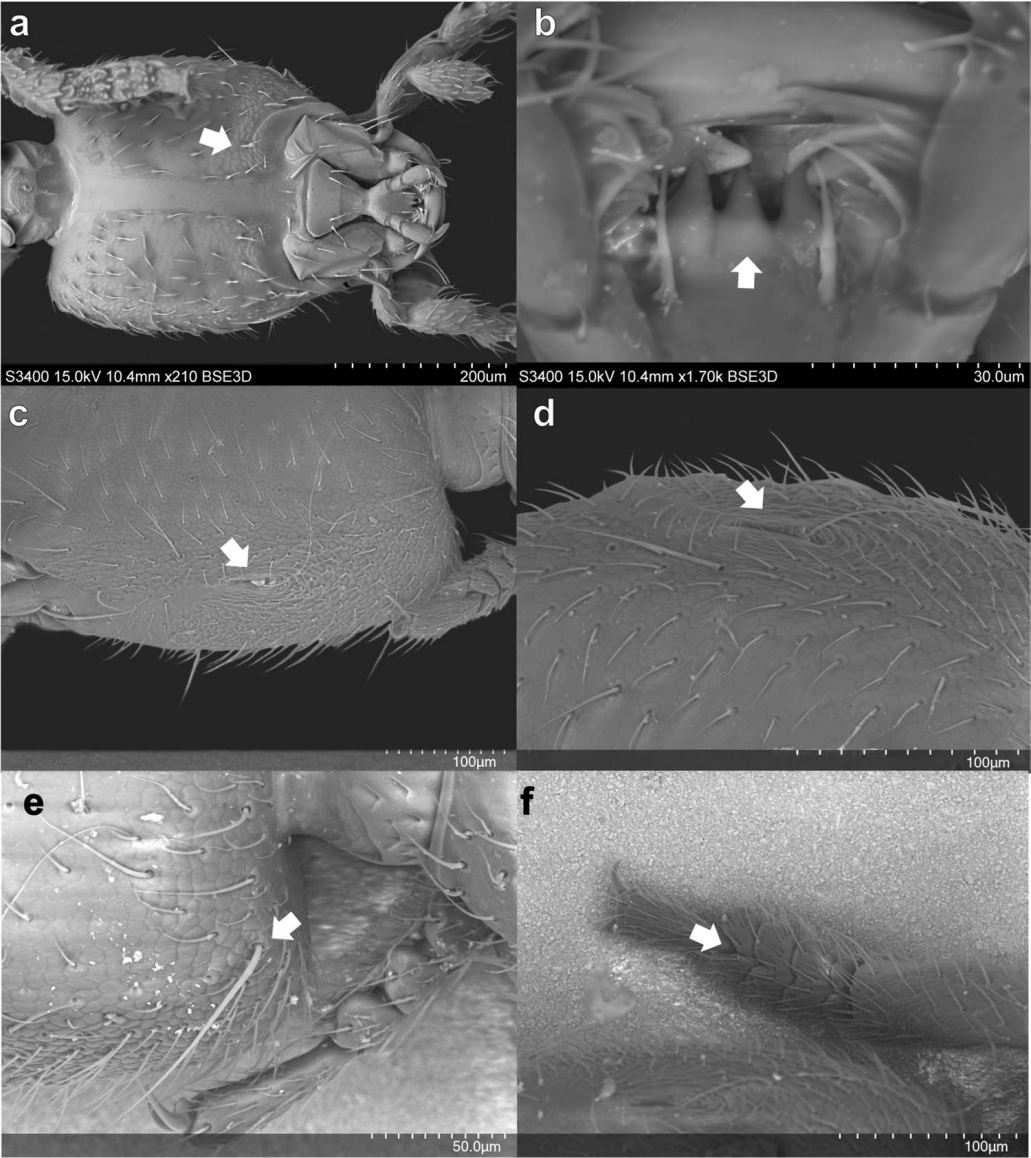
*Micranops bolivianus* Guzman & Żyła **sp. nov. a–e** Head. **a** Head ventral view. microsculpture (arrow). **b** Ligula trilobed (arrow), ventral view. **c** Trichobothrium on the lateral side, cavity of trichobothrium (arrow). **d** Close-up of trichobothrium, cavity of trichobothrium (arrow). **e** Posterior margin, thick seta (arrow). **f** Forelegs, bilobed tarsomere 4 (arrow)

Figures 1a–i; 2 a–f; Online Resource 1.

urn:lsid:zoobank.org:act:EF82A1B8-EA19-451B-8755-40AE27250E55.

**Diagnosis.** *M. bolivianus* Guzman & Żyła **sp. nov.** differs from other Neotropical *Micranops* by the lack of eyes (eyes in *M. brunneus* are rudimentary, but still present). In the structure of the aedeagus, it resembles *M. surinamensis*, but the apex of the ventral process is wide and blunt, while in *M. bolivianus* Guzman & Żyła **sp. nov.,** it is thinner and pointy. From all eyeless species worldwide, it resembles *M. mlejneki* Frisch and Oromí, 2006 in the structure of aedeagus. It differs by the ventral process thin and longer than the median lobe (Fig. 1 d–i), while in *M. mlejneki* ventral process is thick and short, not exceeding the median lobe (figs 5–10 in Frisch and Oromí 2006).

**Etymology** The species name is derived from the country of the species type locality—Bolivia. An adjective.

**Material examined. HOLOTYPE** BOLIVIA; ♂; “*M. bolivianus* Guzman and Żyła 2023 HOLOTYPE [red label] \\ BOL/Nov2013/09 **BOLIVIA 2013, SANTA CRUZ dep., Comarapa, W Amboro NP Siberia pass** env., 2450 m, 28.xi.; S17°50′12.0" W64°42′08.0" Sifting litter Winkler app extr.; P. Baňař lgt.; \\ COLLECTION; P. BAŇAŘ; Moravian Museum Brno”. (UASC); **PARATYPES** BOLIVIA; 5 ♂; 2 ♀; PARATYPE [yellow label] \\ ♀; “ BOL/Nov2013/12 **BOLIVIA 2013 SANTA CRUZ dep., W Amboro NP Barrientos**, 1813 m, 29.xi.; S18°06′0.6" W63°48′08.0" Winkler app. extr.; P. Baňař lgt. \\ COLLECTION; P. BAŇAŘ; Moravian Museum Brno”; 5 ♂ and 1 ♀; same data as for holotype. (3 ♂, 1 ♀ MMBC; 1 ♀ UASC; 1 ♂ JJRC; 1 ♂ ZMH).

### Description

*Measurements* (n = 1, Holotype), FBL (1.16), H (0.38, 0.33), A (0.62), a1 (0.09, 0.06), a2 (0.07, 0.04), a3 (0.05, 0.03), a4 (0.05, 0.03), a5 (0.05, 0.04), a6 (0.05, 0.04), a7 (0.05, 0.04), a8 (0.04, 0.04), a9 (0.05, 0.05), a10 (0.04, 0.05), a11 (0.07, 0.06), NKW (0.12), GL (0.04), P (0.4, 0.3), E (ext 0.3, int. 0.22, 0.32), AB (1.3), PC (0.2, 0.07), PF (0.3, 0.14), PT (0.2, 0.05), MSC (0.15, 0.08); MSF (0.23, 0.8), MST (0.2, 0.04), MTC (0.10, 0.12), MTF (0.3, 0.12), MTT (0.3, 0.04).

*Colouration.* Unicolourously brown, appendages and labrum lighter, mandibles darker brown.

*Body.* Total length 2.45 mm, forebody length 1.2 mm (Fig. 1a); body surface with dense pubescence and dense isodiametric microsculpture resembling scales (Fig. 2f), more notable on head and pronotum.

*Head.* Head capsule oblong, 1.2 times as long as wide; anterior margin straight, elevated, with two protruding processes above antenna insertion, each process with two thick setae, one long above and one short at sides (Fig. 1a). Eyes absent. Gena slightly invaginated anteriorly close to antenna insertion one pair of trichobothria placed on middle of lateral margin, with wavy surface around cavity (Fig. 2 c–d), rounded towards posterior margin; posterior margin of head slightly emarginate across neck incision, rounded laterally, with two longer and thicker setae close to posterior angles of head (Fig. 2e). Epicranium with scale-like microsculpture, dense setation laterally, scarcer medially. Gula with glabrous surface between gular sutures and dense isodiametric macrosculpture, setation evenly distributed on lateral sides, invagination on anterior margin on both sides of gula sutures. Neck with two central pits joined anteriorly. Antenna moniliform, longer than head, shorter than head and pronotum combined, antennomere 1 as long as antennomeres 2 and 3 combined, antennomeres 2–9 longer than wide, antennomere 10 wider than long, antennomere 11 longer than wide. Mandibles tridentate. Labrum transverse, bilobed, with acute processes on each lobe and deep emargination between lobes. Maxilla, palpomere 1 slightly expanded apically, glabrous; palpomere 2 elongated, widened apically, as long as palpomere 3; palpomere 3 globular, narrowed posteriorly, with setation; palpomere 4 minute, conical and glabrous (Fig. 2a). Stipes with three well-developed setae on lateral margin and two at base. Cardo with one central seta. Galea and lacinia with microsculpture. Labium, palpomere 1 quadrate; palpomere 2 longer than 1, slightly wider apically, with setation on distal border; palpomere 3 thin and glabrous. Ligula trilobed, with lobes broad apart; mentum with four central setae, arranged in pairs. Submentum with one seta on each side (Fig. 2b).

*Thorax.* Pronotum 1.3 times longer than wide, with maximum width in middle of length; oval, as long as head, surface covered with setae, disc with longitudinal invagination in middle. Anterior margin strongly convex. Posterior margin truncated. Prosternum with microsculpture, basisternum transverse, without transversal or longitudinal carina (fig 4, Bogri et al. 2020, Online Resource 1, photo with habitus ventral side). Furcasternum with longitudinal carina. Basisternum with microsculpture, transversal carina poorly defined. Metasternum with microsculpture, intercoxal process with one pair of rounded processes. Elytra distinctly shortened, as long as wide, surface shiny, elytron with epipleural ridge; apical angle rounded, costal side longer than anal, fringed with row of setae.

*Legs.* Procoxa 2.8 times longer than wide; profemur widest at middle, twice as long as wide; protibia with 4 combs, wider combs located distally; protarsomeres 1–4 wider than long, with short and dense setae inner side, bilobed (Fig. 2f). Mesocoxa twice as long as wide; mesofemur 3 times longer than wide (0.23, 0.08), mesotibial ctenidium subequal on both sides; mesotarsomeres 1–4 subequal and mesotarsomere 5 longer, elongated and cylindrical. Metacoxa as long as wide; metafemur widest in middle, twice as long as wide; metatibial ctenidium on both sides, inner longer than wide; metatarsomeres 1–4 subequal and 5 longer, elongated and cylindrical.

*Male abdomen.* With microsculpture, setation short on surface, longer row on anterior margin; tergite III–XII anterior margin straight, tergites IX–XI fused and posterior margin rounded (Fig. 1c); sternite VIII with U-shape central emargination about 1/3 of sternite length (Fig. 1b).

*Aedeagus.* Teapot shape; median lobe wider at base, apex with rounded process projecting on parameral side, with scarce micropunctuation (Fig. 2d); ventral process thin and longer than median lobe, narrowing towards apex (Fig. 1 d–i).

*Female abdomen.* Usually lack of emargination on abdominal segments, sometimes present, but shallow, always shallower than in male.

**Fig. 3:**
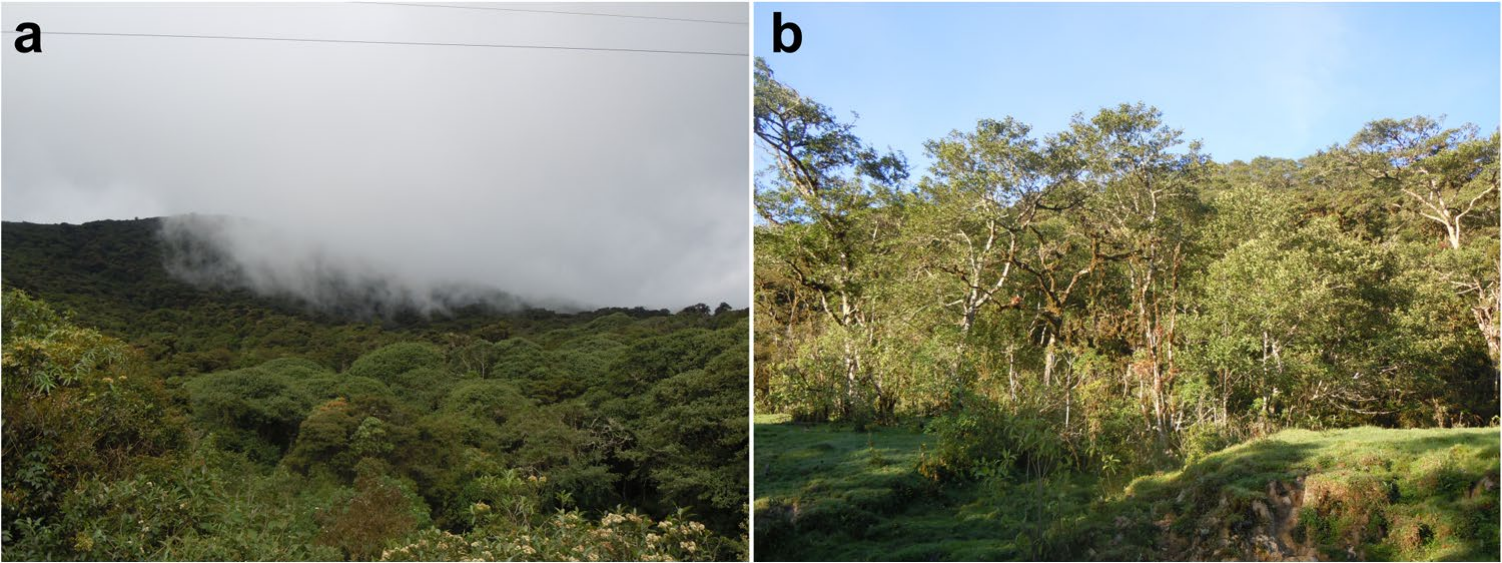
Habitat of *Micranops bolivianus* Guzman & Żyła **sp. nov.**, Parque Nacional Amboró, Santa Cruz, Santa Cruz de la Sierra. **a** Cloud forest. **b** Vegetation in the type locality. Photo credits Petr Baňař

**Distribution** The species is known only from the type locality in Amboró National Park, Comarapa, Santa Cruz province, Bolivia. It was collected at a high altitude of 2450 m.a.s.l. in the locality called “Siberia”, a cloudy forest (Fig. 3), by sifting leaf litter and using a Winkler extractor.

### Additional records of *Micranops*

*M. cf. surinamensis* (Herman 1965).

SURINAME; ♀; “XVIII; Vau k. leg; 8.59 \\ MUSEUM PARIS; 1983; Coll H. COIFFAIT”; ♀; “VIIIa; Taubahredjo; 6.59 \\ MUSEUM PARIS; 1983; Coll H. COIFFAIT”. MNHN.

Note: Based on observation of the posterior margin of the sternite VIII and IX and comparison with (fig 13. in Herman (1965)) for *M. surinamensis* and its geographic distribution, we suggest this specimen may correspond to a female of the species *M. surinamensis.* Sternite VIII has a medial light emargination with a longitudinal depression, while sternite IX has a mid-deep emargination. However, we do not have enough evidence to undoubtedly classify it as conspecific.

*M. cf. chloroticus* (Sharp 1876).

BRAZIL; 4 ♀; “Umg. Manaus; Amazonas geblet; Brasil; Ig. L. Beck leg\\ lago da na#aca [hand writing] \\MUSEUM PARIS; 1983; Coll H. COIFFAIT”. MNHN.

Note: Based on the diagnosis and description in Sharp (1876), and its geographic distribution, we suggest this specimen may correspond to a female of the species *M. chloroticus*. The common features are as follows: predominantly yellowish body colouration, head longer than wide, with parallel lateral margins and rounded posterior angles, prothorax longer than wide, sides parallel, anterior angles rounded, and elytra clearly wider and a little longer than thorax, very pale yellow, with micropunctures barely visible. However, we do not have enough evidence to undoubtedly classify it as conspecific.

*Micranops* spp. Cameron 1913

We report a new country record for Ecuador in the provinces of Cotopaxi, Guayas, Imbabura and Pichincha, and a new record for Peru in the Cusco region.

With eyes

ECUADOR; ♀; “Pichincha, Near Tocachi, 50 m a.i of Rio Pilation [0°18′57.7"S, 78°57′17.1"W]\\ grasscover suceeding clear-cutting; 19.II.1986; A. Zicsi & I. Loksa leg.” HMNH.; 5 ♀; “ Ingenio San Carlos; b. Guayaquil; VIII.1975; Ecuador; H. Franz leg\\MUSEUM PARIS; 1983; Coll H. COIFFAIT”. MNHN.

Eyeless

ECUADOR; 1 ♀; “Imbabura, 30 km from Otavalo to Apuela, Otocique [0°20′21.2″N 78°24′41.0″ W]; 3250 m, paramo \\ Couch-grass, soil and litter; 19.IV.1989; A. Zicsi & I. Loksa leg.; (B87)”; ♀; “ Cotopaxi, Cotopaxi National Park, Paramo [0°39′24.5″S, 78°22′23.2″W]; 4000 m, \\ moss from the bank of a water gully; 26.II.1986; A. Zicsi & I. Loksa leg.; (Berl204)”; ♀; “Imbabura, above Otavalo [0°14′03.1″N 78°15′41.2″W]; 3350 m \\ cut-over area; 19.IV.1989; A. Zicsi & I. Loksa leg.; (B84)”; ♀; “Imbabura, 52 km from Otavalo [0°14′03.1″N, 78°15′41.2″W]; 3350 m \\ waterfall, moss and soil from rocks; 20.IV.1989; A. Zicsi & I. Loksa leg.; (B98)”; ♀; Pichincha, Pasochoa Nat. Park, 50 m above the creek [0°27′34.2″S 78°27′08.4″W]; 2800-2850 m \\ *Rubus* shrubs litter; 6.II.1986; A. Zicsi & I. Loksa leg.; (Berl34)”. HMNH.

With reduced eyes:

PERU; ♀; “18.x.2002; MACHU PICCHU; ca. 2400 m, forest; J. Janák lgt., sifting”. JJRC.

## Discussion

**Fig. 4:**
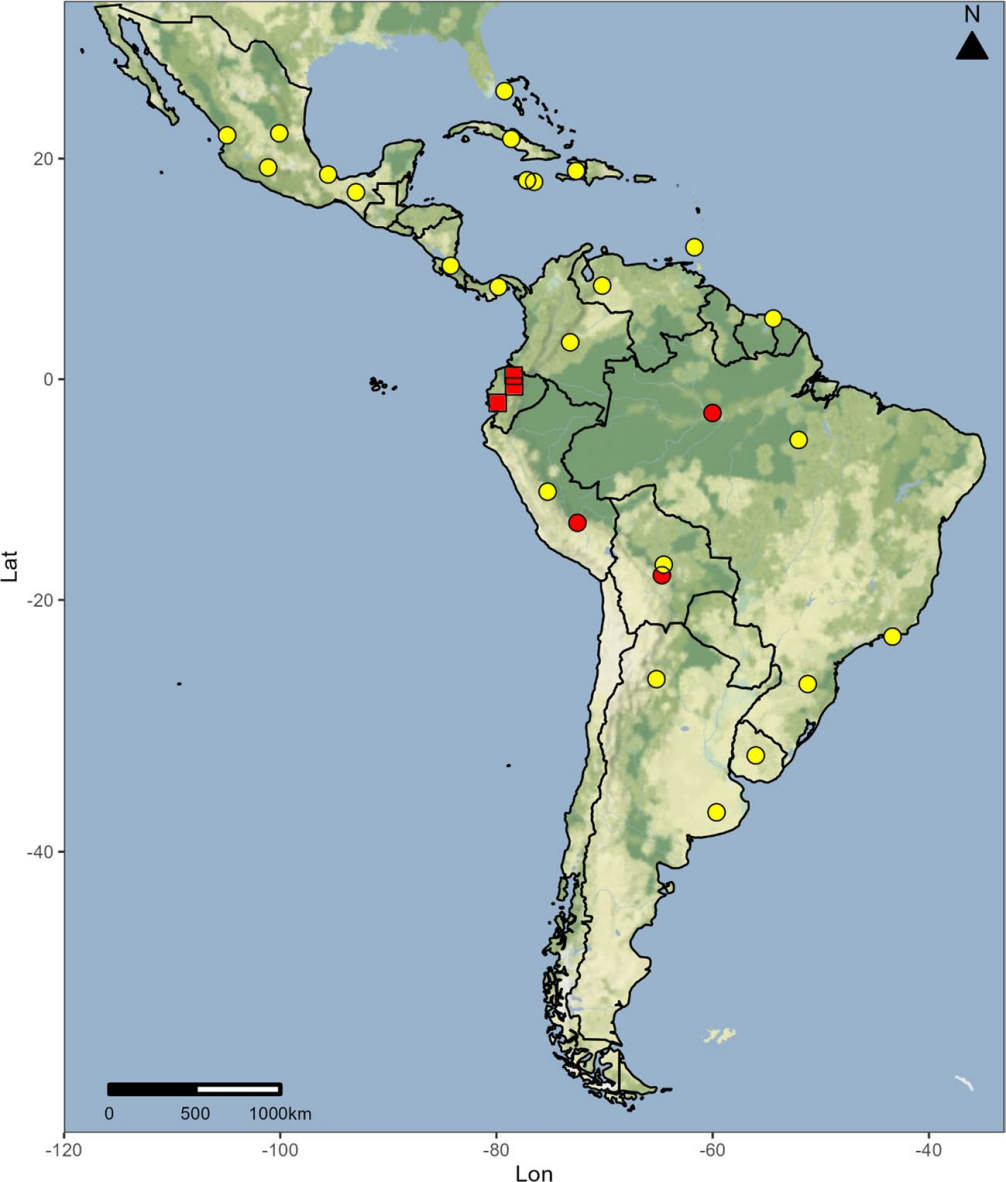
Occurrences of *Micranops* spp. in the Neotropics; new locality records (red dots), a new country record (red squares), previous records after Frisch and Herman 2014 and Herman 2023 (yellow dots)

We have described a new blind species of *Micranops*, *M. bolivianus* Guzman & Żyła **sp. nov.**, from the Neotropics, increasing the number of known species for this region to seven. Additionally, we report new genus occurrences in the western Andes mountain range, thus expanding its distribution range to the western part of South America (Fig. 4). In the worldwide fauna of *Micranops*, the absence of eyes is observed in species like *M. spelaeus* Frisch and Oromí, 2006 from Canary Islands caves, or *M. subterraneus* Frisch and Oromí, 2006 collected deep in the gravelly soil, which is believed to be an adaption to the troglobitic and endogean way of life (Frisch and Oromí 2006). In the case of *M. bolivianus* Guzman & Żyła **sp. nov.**, it was collected by sifting leaf litter and using a Winkler extractor; however, the specific depth of occurrence remains unknown, preventing us from undoubtedly associating the absence of eyes in this species with endogenous habitat. While these characteristics are commonly associated with such a way of life, no conclusive evidence supports the notion that these traits are adaptations conferring an advantage with differential reproduction. Further studies employing comparative methods of character mapping on the phylogeny and techniques such as microtomography that can show new connections in optical nerves, among others, could shed light on this intriguing question.

### Electronic supplementary material

Fig. S1 ESM 1(PNG 1.72 mb)

High resolution image (TIF 22.6 mb)

## Data Availability

All the data used and findings of this study are included in in the article.
