# Supplementary figures and images for: A New Eyeless Species of Micranops Cameron 1913 from Bolivia (Coleoptera: Staphylinidae: Paederinae)
Source: Neotrop Entomol. 2023 Dec 14;53(1):154–61. doi: 10.1007/s13744-023-01106-5 (PMC10834603; doi:10.1007/s13744-023-01106-5)

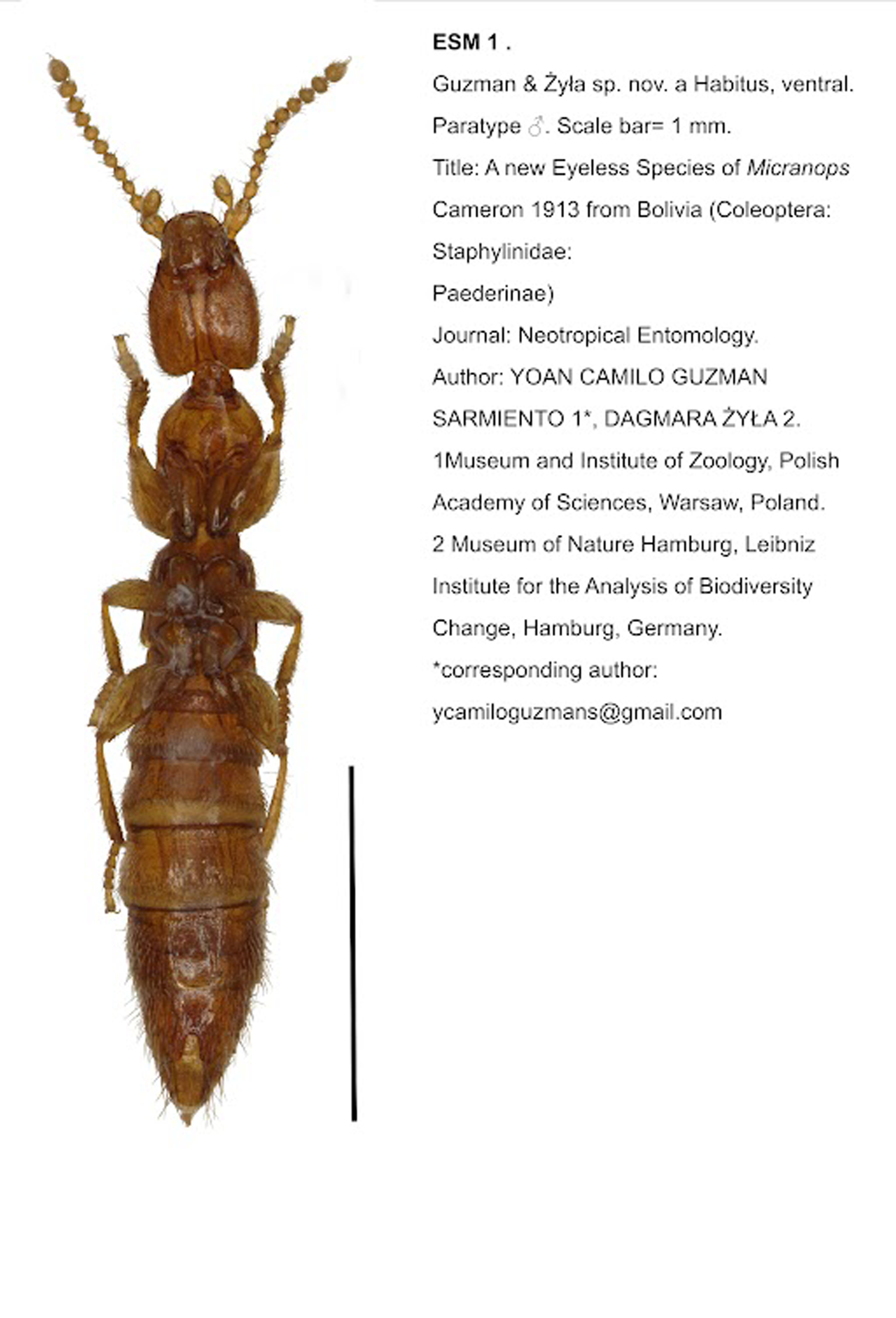

Supplement: Supplementary file 1 — ESM 1(PNG 1.72 mb) [file 13744_2023_1106_Figa_ESM.png]

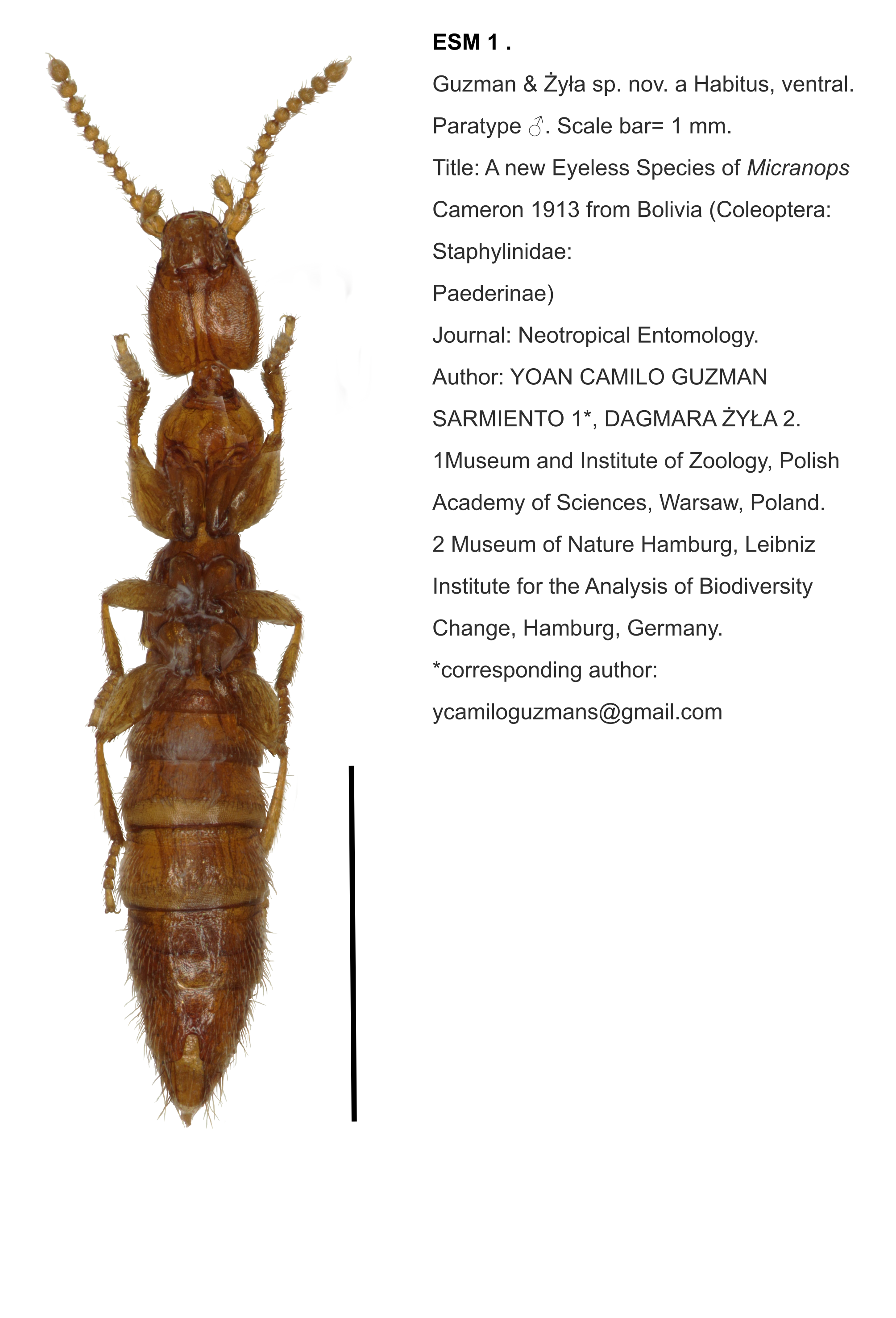

Supplement: Supplementary file 2 — High resolution image (TIF 22.6 mb) [file 13744_2023_1106_MOESM1_ESM.tiff]
